# Creativity Alone Does Not Make a Star – Social Attributes of the Nomination of Creative Icons: Results of a Trend Study in Germany

**DOI:** 10.3389/fpsyg.2018.01944

**Published:** 2018-10-16

**Authors:** Min Tang, Christian H. Werner, Sebastian Hofreiter

**Affiliations:** Institute for Creativity & Innovation, University of Applied Management, Ismaning, Germany

**Keywords:** implicit theory of creativity, aesthetic salience, meritorious salience, Germany, trend study, serial multiple mediation model, three-path mediation model

## Abstract

Recent years have witnessed a series of studies of the nomination of the most creative persons using a cross-sectional design. Such studies only provide a “snapshot” of the creativity nomination phenomenon without being able to detect the temporal pattern of the nomination over time. The current study is among the first of such studies that use a time series design. Data were collected from German young adults in 2013 (*n* = 460, *M*_age_ = 28.3, *SD* = 9.9) and in 2017 (*n* = 617, *M*_age_ = 31.4, *SD* = 10.6). Consistent patterns emerge from the nomination of the top 10 most creative Germans: (1) Artists are predominantly represented; (2) Male creators are predominantly nominated; (3) Einstein ranks the first in both lists followed by Goethe; (4) Merkel is the only female nominee in both lists. Analysis of all nomination in both years reconfirmed the aesthetic salience and male-dominance and these patterns were more likely to occur in earlier than later nominations. Regression analysis revealed that social contribution (SC) and social acceptance (SA) each mediated the positive relation between creativity and creative fame. Further, the three-path mediation model of creativity on creative fame through SC and SA was also significant for both nomination conditions, with stronger mediating effect on the nomination from the meritorious than the aesthetic areas. Domain-specificity theories and social psychological theories were used to interpret the results.

## Introduction

Modern sociocultural theories of creativity maintain that creativity is not only a personal but also social construct, which involves an interaction of multiple factors in and outside the person (for a review see Tang, 2017). Hence, creativity can be optimally examined only if both the individual and environmental variables are taken into account. This approach is of particular value to the studies of implicit theories of creativity, as the opinion formation of laypersons are particularly susceptible to social influence (Moussaïd et al., 2013).

Up to now, the majority of research on implicit theories of creativity primarily focuses on the perceptions of attributes of creativity from the laypersons’ point of view (e.g., Runco, 1989; Runco et al., 1993; Lim and Plucker, 2001; Seng et al., 2008). Another stream of studies investigate the perceptions of creative representatives and revealed the *meritorious* vs. *aesthetic salience* concerning students’ nominations of creative individuals (e.g., Yue and Rudowicz, 2002; Yue, 2003, 2004; Cheung and Yue, 2007). Meritorious salience places more emphasis on the creators’ SCs and influence, whereas aesthetic salience underscores the novelty and individuality of the creative persons. Several studies conducted with Chinese samples have found an obvious meritorious salience in the Chinese nomination of the creative representatives (e.g., Yue, 2003, 2004; Cheung and Yue, 2007). In contrast to this, the aesthetic salience has been more theorized than empirically tested, except two recent studies of Yue et al. (2011) and Tang and Moser (2018), which involved German samples. Both studies, and the all above-mentioned studies, all used a cross-sectional design, which could only provide a “snapshot” of the creativity nomination phenomenon without being able to detect the temporal pattern of the nomination over time. The current study is, to our knowledge, the first that uses a time series design to detect the nomination of creative persons among German young adults across time. Focus of this trend study are the patterns of Germans’ nomination of creative icons and the personal and social attributes of such nomination.

### Implicit Theories of Creativity (ITC)

Implicit theories are explanations held by laypersons (such as students, teachers, and parents) for particular psychological phenomena or constructs (Sternberg, 1985). Sternberg (1985) pointed out that studies of implicit theories are of theoretical and practical importance for complex constructs such as creativity. Since 1980s, more and more studies have been taken to explore implicit theories of creativity of different layperson groups including teachers and/or parents (e.g., Runco, 1989; Runco et al., 1993; Chan and Chan, 1999; Runco and Johnson, 2002; Seng et al., 2008), students (e.g., Seng et al., 2008; Karwowski, 2009), or politicians, scientists, artists, and school teachers (Spiel and von Korff, 1988) or large samples with people of different age and professional groups (Lim and Plucker, 2001). These studies share the fact that they primarily focus on the perceptions of the concept and nature of creativity from a layperson’ point of view and apply a social validation method to examine the naïve beliefs of creativity. Studies of ITC of recent years further blossom by combining and comparing the views of different cultures (e.g., Runco and Johnson, 2002; Paletz and Peng, 2008; Yue et al., 2011; Lan and Kaufman, 2012), detecting the relationship of ITC, self-beliefs and domain (Hass et al., 2017), the implicit theories of creativity and intelligence (Plucker et al., 2017) and laypersons’ perceptions of creativity symbols (Glãveanu, 2011).

Another stream of studies, mainly conducted in China, focus on Chinese students’ perceptions of creative icons, both domestic and international (e.g., Yue and Rudowicz, 2002; Yue, 2003, 2004; Cheung and Yue, 2007). Through these studies, they have consistently observed that Chinese students lay more weight on SCs, meritorious service, recognition, influence, and the utilitarian practice of creative individuals in their perceptions of creative persons and tend to nominate politicians, scientists, inventors, businessmen, and strategists as creative icons (Yue and Rudowicz, 2002; Yue, 2003, 2004; Cheung and Yue, 2007). They called this pattern of nomination the *meritorious salience* evaluation (Yue, 2003; Yue et al., 2011). In contrast, Western people emphasize more liberal individualism, freedom of expression, self-actualization, and ideas of equality (Spinks et al., 1998; Lubart, 1999; Dineen and Niu, 2008) and tend to nominate artists, writers, and philosophers as representatives of creators (Yue et al., 2011). They called this pattern of nomination the *aesthetic salience* evaluation. This hypothesis was confirmed by an intercultural study involving 437 Chinese and 166 German undergraduates (Yue et al., 2011) which found that while Chinese undergraduates nominated more politicians, scientists, or inventors, but rarely artists and musicians, the German undergraduates mostly nominated philosophers, artists, and writers but rarely politicians as their creative icons. However, this study only described the results of the nominations, but did not examine the underlying process which might attribute to specific nominations. A recent study using a German student sample was able to reconfirm the aesthetic salience in the German style of nomination (Tang and Moser, 2018). This study also discovered that aesthetic salience could be partly explained by a four-factor creator evaluation model composed of creativity, liking, SCs, and SA. The results of this study point to the important roles sociocultural factors play in the nomination of creative icons among Western people such as Germans.

### Sociocultural Theories of Creativity

Nobody is living in vacuum. Society is the place where we develop into social persons through interaction with others. In society, individuals usually rely on the observation of others to adapt their behaviors, revise their judgments, or make decisions (Couzin et al., 2011). Creative and prominent people are usually famous people who have won recognition by a large number of people (Schwartz, 1998). A creator is more likely recognized when he or she has exerted personal influence over others and has attracted admirers of his time (Simonton, 1988). This means that not only personal attributes (such as creativity and originality) but also social attributes such as (SCs and SA) are important determinants of the evaluation of creative persons. Sociocultural approach to creativity examines creativity by taking into the consideration of social systems where creativity occurs and is evaluated (Csikszentmihalyi, 1994; Amabile, 1996). Social experience is the basis on which people develop their theories about their own creativity and the creativity of others (Hass et al., 2017). Considering this, it seems imperative to approach the issue of creativity judgments from a sociocultural perspective.

In their studies, Yue and Rudowicz (2002), Yue (2003), and Cheung and Yue (2007) asked the participants to list the reasons why they nominated certain creators. Analysis of the justifications revealed that the factors that led to the nominations were not restricted to the dispositional attributes of the creators such as creative expressions and originality. Rather, they also covered social factors such as the fame and SCs of the nominated persons. They found that Chinese students attached more importance to SCs in nominating creators from the meritorious salience fields and more importance to creativity level in nominating creators from the aesthetic salience fields (Yue, 2003). The above results were found among Chinese students. The current study attempts to examine the issue with German samples.

### Gender Stereotypes and Creativity

Researchers have been long interested in the relation between gender and creativity. Although anecdotal evidence tends to suggest a clear disparity in creativity in favor of males, narrative reviews and meta-analyses have not provided adequate evidence to support this. One of the first review about gender and creativity was published by Kogan (1974). Over three decades later, Baer and Kaufman (2008) updated Kogan’s study by analyzing a wide range of studies involving different age groups and using different creativity tasks (e.g., creativity test scores, creative achievements, and self-reported creativity). Like Kogan (1974), they did not find obvious gender differences in creativity. Furthermore, the handful studies that revealed significant gender differences in their review suggested a slight lead of females than males in creativity. Interestingly, this slight female-lead in creativity has been reconfirmed by two recent meta-analysis studies based on large samples. Ma (2009) meta-analyzed 2,013 effect sizes of 111 studies about the relevant variables associated with creative person, process, product, and environment and found an almost negligible lead of females in creativity level (*k* = 104, mean effect size = 0.14, *SD* = 0.43). Thompson’s (2016) meta-analysis involving 271 studies, 137,247 participants, and 480 independent effect sizes discovered a weak though significant relationship between creativity and gender (*r* = 0.056, *p* < 0.05) suggesting a slight female superiority in creativity than males.

Although empirical studies provide much evidence to the lack of male superiority in creativity, actual achievement leaves little room for debate. Historically females have been underrepresented among recognized high-achieving creators, inventors, and innovators (Tang, 2010; Thompson, 2016) and a consistent male-dominance in many creative fields can be observed (Simonton, 1994; Piirto, 2004). Proudfoot et al. (2015) conducted a series of correlational and experimental studies to detect the association between masculinity and creative thinking. The results of their studies revealed a gender bias in the attribution of creativity favoring males and disregarding females. Similar endeavors of Luksyte et al. (2018) on the basis of three field and experimental investigations found that innovative work behaviors were also stereotypically associated with men and the work done by males were overall more favorably evaluated than the work done by females. This stereotypical perception has also been found in nomination studies. For example, British undergraduates’ predominantly nominated male rather than female geniuses (Smith and Wright, 2000). Cheung and Yue’s (2007) found that male creators were significantly more frequently nominated as the most creative persons, though the qualities of the creativity did not differ between male and female creators. Based on the previous studies, it is hypothesized that more male than female creators will be nominated in both studies.

### Availability and Affect Heuristic in Judgments

Nomination of creative persons is a typical judgment and decision-making process, which involves the interplay of cognitive and affective heuristics. Two most influential heuristics related to this process are the availability and affect heuristics. Availability heuristic is defined as the process of judging frequency or probability of events “by the ease with which relevant instances come to mind” (Tversky and Kahneman, 1973, p. 207). In making social judgments, individuals typically tend to rely on a subset of information most accessible from memory instead of searching exhaustively in the memory for information supporting the judgment (for reviews, see Sherman and Corty, 1984). In one of their studies, Tversky and Kahneman (1973) presented the participants with a list consisting of names of famous and less famous names and one task for the participants was to recall the names. Because famous names are generally easier to recall, they hypothesized that a class consisting of famous names should be judged more numerous than a comparable class consisting of less famous names. Results of the experiment confirmed their hypothesis. Hence, it is expected that more famous people will enjoy higher fame in the current study.

In parallel to the cognitive heuristic, the importance of affect has also be recognized by judgment and decision researchers. Zajonc (1980) pointed it out, “Quite often “I decided in favor of X” is no more than “I liked X…” We buy the cars we “like,” choose the jobs and houses we find “attractive,” and then justify these choices by various reasons…(p. 155).” Slovic et al. (2007) echoed Zajonc by maintaining that people form opinions and make choices that directly express their feelings, because “…readily available affective impression can be far easier – more efficient – than weighing the pros and cons or retrieving from memory many relevant examples, especially when the required judgment or decision is complex or mental resources are limited” (p. 1336). Liking is positive affect, which typically result in approach tendencies to persons or objects. Therefore, liking is also considered in the current study and a positive correlation between being liked and the frequency of being nominated can be expected.

The present study focuses on two major questions: Firstly, does the aesthetic salience and male dominance, which was observed in previous studies, hold true for German young adults using a time series design? Secondly, what kind of roles do personal (creativity and personal liking) and social attributes (SC and SA) play in Germans’ nomination of creative representatives?

## Materials and Methods

### Participants

A total of 460 German young adults (57.8% females, *M*_age_ = 28.3, *SD* = 9.9) participated in the first study in 2013. The youngest was 18 and the oldest 65 years old with the majority (85.2%) younger than 35. Among them, 185 (40.2%) were students and the rest employees. The students were from different subject areas, including 47.6% majoring in social sciences or economics, 12% in physics or engineering, 9% in humanities, and 6.5% in arts. Over half (51.6%) of the employee participants were working in the social sciences, economics or health areas, 24.4% in the technical or information areas, and 3.3% in the artistic areas. On average, the employee participants had worked 10 years (*SD* = 10.8) with an average weekly working hours of 36.8 (*SD* = 12.4).

In 2017, the number of the participants increased to 617 (59.8% females, *M*_age_ = 31.4, *SD* = 10.6). The youngest was 18 and the oldest 68 years old with the majority (78.4%) younger than 35. Among them, 260 (42.1%) were students, 300 (48.6%) were employees, and 40 were free-lancer (6.5%). The students were from different subject areas, including 15.2% majoring in economics or law, 7.9% in social sciences, 7.8% in physics and engineering, and 1.8% in arts. Of the employee participant, 34.7% were working in the social sciences, economics, or health areas, 10.2% in the technical or information areas, and 2.8% in the artistic areas. The employee participants had an average working experience of 11.4 years (*SD* = 10.2) and were working 39.8 h (*SD* = 10.5) per week. On the whole, the sample of Study 2 is comparable to that of Study 1 in terms of the proportions of age, gender, occupation, and study or work areas, etc.

### Measures

#### Areas of Creative Achievement

The nomination was measured through a standardized questionnaire in which the participants first nominated up to three most creative Germans, then chose the area in which the nominated person has made the major creative contributions. The areas used in this study was a combination and slight adaptation of the categories used in previous studies (Cheung and Yue, 2007; Yue et al., 2011), which included ten areas: (1) scientists/inventors (including scientists, inventors, doctors, engineers, architects); (2) politicians (including emperors, ministers, governors, heads of state, social, or human rights activist); (3) writer/poets (including authors, poets, novelists, essayists); (4) philosophers/educators (including philosophers, educators, scholars, religious thinkers); (5) fine artists (including painters, draftsmen, photographers, architects, ceramists, conceptual artists); (6) performing artists (including musicians, composers, singers, dancers, actors, entertainers); (7) generals/military strategists (including generals, military strategists, military theorists); (8) businessmen/entrepreneurs (including financial managers, bankers, business managers); (9) sportsmen/coaches; and (10) Others (e.g., fictitious or godly figures). If a nominee was creative in more than one domain, the person would be coded by what he or she was best known for, as agreed by the two coders. Inter-coder consistency was high, with kappa coefficient of 0.93 and 0.95 in 2013 and 2017, respectively.

The creative achievement areas were subdivided into three groups, including aesthetic salience areas (arts, literature, and philosophy/education) and meritorious areas (science/invention, politics, business/entrepreneurship, cooking, and military) (Yue, 2003; Yue et al., 2011), and others (sports and non-celebrities whose fields of achievement cannot be identified). It’s worth noting that though it is pervasive to regard cooking as a kind of art (“culinary art”), there seems to lack empirical evidence to this classification. For example, Kaufman Domains of Creativity Scale (2012) classified creativity in cooking to everyday creativity in parallel to other domains such as performance and arts. Carson et al. (2005) Creative Achievement Questionnaire, which measures creative achievement in 10 different domains discovered that “culinary art” falls into the science and invention category instead of arts. Therefore, cooking is put to the meritorious salience category where science and invention also belong too.

#### Creativity and Social Contribution: Study in 2013

The justification of the nomination was measured somewhat differently in 2013 and 2017. In 2013, the two questions from the previous study (Cheung and Yue, 2007) were applied. These two questions were: (1) How creative is this person? (3) How much does the creator contribute to society? Both questions were measured with a 10-point Likert scale ranging from 1 = “the least” to 10 = “the most.”

#### Creativity, Social Contribution, Liking, and Social Acceptance: Study in 2017

In 2017, a 13-item Creator Evaluation Scale (CES; Tang and Moser, 2018) was applied to examine the factors behind the nomination in a more thorough way. The CES was developed on the basis of the three key dimensions that Cheung and Yue’s (2007) study has identified, namely creativity, SC, and esteem. Esteem was revised into liking, because liking is an important affect heuristic that people usually reply on in making judgments and decisions (Slovic et al., 2007). Besides, liking is also a typical criterion in creativity evaluation studies (see Amabile, 1996). A fourth dimension, namely “SA” was added, because SA has been widely used to help explain behavior, opinions and beliefs in sociology, marketing, and political science, etc. (see Sewell, 2018). SA in this study is similar to the construct of “felt social norms” which refers to a person’s perception of the encouragement or discouragement of his/her significant others with regard to a certain behavior (Ajzen and Fishbein, 1980). The current study is not about behavior decision but people’s subjective individual evaluation of creative persons, therefore “norms” is not an appropriate term. The items focus on measuring to which degree the nominated person is popular, recognized, and valued by the significant others of the participants and society. Therefore, this variable is named “SA.”^1^

***Creativity*** was measured with three items measuring the creative, original, and insightful level of the person (e.g., “How creative is this person”?). ***Liking*** was measured with three items about how much the participants like, value and admire the person (e.g., “How much do you like this person?). ***Social contribution*** was measured with three items dealing with the question “How significant is the SC of this person.” ***Social acceptance*** was measured with four items about how much the person was popular, recognized, and valued by one’s friends, relatives and the social media (e.g., “How much do your friends esteem this person”?). Participants were asked to give their rating on a 10-point Likert scale ranging from 1 = “the least” to 10 = “the most.” The inter consistencies of the variables are high, with Cronbach’s α of 0.65 for creativity, 0.76 for liking, 0.82 for SC, and 0.78 for SCs. The current study focuses on the nomination of highly creative persons. The four factors of the CES reflect the rationalization why somebody has been nominated. No wonder all four variables demonstrate somewhat negative skewness. Following the advice of Tabachnick and Fidell (2007), all four variables were transformed using the formula “NEWX = SQRT(K-X) (p. 89). As this procedure involved a reflection procedure, results from subsequent analyses were reflected back.

***Creative fame*** was computed by summing the number of nominations made by the participants. This method is similar to the studies of public perceptions (Schwartz, 1998) and consistent with what was applied in the Cheung and Yue’s (2007) study. The fame scores range from 1 to 140 (*M* = 35.7, *SD* = 49.0) in 2013 and from 1 to 124 (*M* = 28.8, *SD* = 40.0) in 2017. In both years, the distribution of the fame scores showed a substantial positive skewness. Hence, this variable was log-transformed for the subsequent data analysis (Tabachnick and Fidell, 2007).

### Procedures

Data was collected via online surveys between July and October 2013 and between April and July, 2017. Participants were treated in accordance with the ethical guidelines set out by the American Psychological Association. They were not rewarded for participating and were informed that they could withdraw at any time. Instruments used in the study in 2013 were adopted and slightly adapted from the study of Cheung and Yue (2007). These instruments, originally in English, were translated into German and back translated into English by two German-English bilinguals with psychological background to guarantee the quality of the translation. In 2017, the Creator Evaluation Scale (CES; Tang and Moser, 2018), which was developed directly in German, was added to the study.

To eliminate the “Google Effects” – the tendency to Google the answers to any possible question instead of making own efforts to find the answers (Sparrow et al., 2011) – two questions were imbedded in the survey to filter out the participants who have used the Internet to look for the most creative Germans. Spelling mistakes in the nominated names were corrected before the frequencies of the nominations were calculated. The focus of the current study is the nomination of the most creative persons of German origin instead of persons from the German-speaking countries; therefore, invalid nominations such as Wolfgang A. Mozart (Austrian), Sigmund Freud (Austrian), Steve Jobs (American), etc., were excluded from data analysis, which resulted in 996 and 1343 valid nominations in 2013 and 2017, respectively.

## Results

### The Top 10 Nominations

**Table 1** presents the top 10 nominated creative persons from Germany in 2013 and 2017. These nominees account for 42.3% (in 2013) and 35.1% (in 2017) of the total nominations. The aesthetic salience is obvious in both studies. In 2013, seven of the top 10 candidates were from the aesthetic salience areas, whereas in 2017, eight of the top 10 candidates were either artists or authors (aesthetic salience areas). In both years, male creators dominated the top 10 list, with Angela Merkel (the current German chancellor) as the only female candidate. Tremendous similarities can be observed from the two lists. The top 4 most creative representatives were exactly the same, with Albert Einstein being ranked the first, Goethe the second, followed by Angela Merkel and Stefan Raab (entertainer and comedian). In parallel to this, five other persons, all artists, appeared in both lists though in somewhat different order.

**Table 1:** The top 10 ranks of the most creative persons from Germany in 2013 and 2017.

	2013					2017					
		
	Rank	*n*	%^a^	Gender^b^	Area of Achievement^c^		Rank	*n*	%^a^	Gender^b^	Area of Achievement^c^
Albert Einstein	1	140	14.1	m	Science (physicist)	Albert Einstein	1	124	9.2	m	Science (physicist)
Johann W. von Goethe	2	86	8.7	m	Literary (poet, writer, naturalist)	Johann W. von Goethe	2	94	7.0	m	Literary (poet, naturalist, playwright)
Angela Merkel	3	50	5.0	f	Politics (current German chancellor)	Angela Merkel	3	51	3.8	f	Politics (chancellor of Germany)
Stefan Raab	4	43	4.3	m	Arts (entertainer, comedian)	Stefan Raab	3	51	3.8	m	Arts (entertainer, comedian)
Ludwig van Beethoven	5	25	2.5	m	Arts (composer, pianist)	Karl Lagerfeld	5	30	2.2	m	Arts (fashion designer, photographer)
Til Schweiger	6	20	2.0	m	Arts (actor, director, producer)	Johann S. Bach	6	28	2.1	m	Arts (composer, musician)
Johann S. Bach	7	18	1.8	m	Arts (composer, musician)	Ludwig van Beethoven	6	28	2.1	m	Arts (composer, pianist)
Friedrich Schiller	8	16	1.6	m	Arts (composer, musician)	Jan Böhmermann	8	25	1.9	m	Arts (satirist, comedy writer)
Karl Lagerfeld	9	12	1.2	m	Arts (fashion designer, photographer)	Friedrich Schiller	9	21	1.6	m	Arts (poet, philosopher, playwright)
Konrad Adenauer	10	11	1.1	m	Politics (first German chancellor	Til Schweiger	10	20	1.5	m	Arts (actor, director, producer)


*^*a*^The percentages are calculated based on the total of 995 and 1343 valid nominations in 2013 and 2017, respectively; ^*b*^m, males; f, females; ^*c*^In case of eminence in multiple areas, only the major area of achievement are listed.*

So far, the analysis was focused on the top rankings and the aesthetic salience was confirmed by both lists. What is the case with the total nomination? Is aesthetic salience still pertinent when all the nominees are taken into consideration? As a whole, are males more frequently nominated than females? To answer these questions, all nominees were combined for data analysis and the results are presented in **Table 2**.

**Table 2:** Total nominations in terms of aesthetic vs. meritorious salience fields, gender, and the nomination order in 2013 and 2017.

	2013								2017							
		
	Nomination and rank^a^			Gender^b^		Nomination order^a^			Nomination and rank^a^			Gender^b^		Nomination order^a^		
						
	Rank	*n*	%	m (%)	f (%)	1 (%)	2 (%)	3 (%)	Rank	*n*	%	m (%)	f (%)	1 (%)	2 (%)	3 (%)
***Aesthetic salience fields***																
Performing arts	1	288	28.9	90.6	9.4	30.5	30.5	24.6	1	455	33.9	88.1	11.9	35.2	34.0	31.6
Literary arts	3	174	17.5	94.3	5.7	17.4	17.8	17.2	3	180	13.4	95.0	5.0	13.7	14.3	11.8
Visual arts	5	64	6.4	93.8	6.3	8.7	4.8	4.9	5	129	9.6	93.8	6.2	11.9	9.3	6.2
Philosophy/education	8	15	1.5	93.3	5.7	1.5	1.0	2.2	9	23	1.7	87.0	13.0	0.9	1.4	3.5
(M)/Sum	(4.3)	541	54.3	(93.2)	(6.8)	58.1	54.1	48.9	(4.5)	787	58.6	(91.0)	(9.0)	61.7	59.0	53.1
***Meritorious salience fields***																
Science/invention	2	238	23.9	99.2	0.8	27.4	21.9	20.9	2	222	16.5	99.5	0.5	19.2	14.0	15.0
Politics	4	142	14.3	61.0	39.0	10.7	16.2	17.5	4	152	11.3	63.2	36.8	10.1	12.1	12.4
Sports	6	42	4.2	95.2	4.8	1.0	5.4	7.8	6	77	5.7	90.9	9.1	2.2	6.7	10.6
Business/entrepreneurship	7	21	2.1	81.0	19.0	2.2	1.9	2.2	8	41	3.1	90.2	9.8	2.6	3.1	3.8
Military/strategy	10	3	0.3	100.0	0.0	0.0	0.3	0.7	11	5	0.4	100.0	0.0	0.3	0.5	0.3
Cooking	11	3	0.3	100.0	0.0	0.2	0.0	0.7	10	7	0.5	100.0	0.0	0.3	0.7	0.6
(M)/Sum	(6.7)	449	45.1	(89.4)	(10.6)	41.5	45.7	49.8	(6.8)	504	37.5	(90.6)	(9.4)	34.7	37.1	42.7
***Others*** (Non-celebrities)	9	6	0.6	33.3	66.7	0.5	0.3	1.1	7	51	3.8	75.0	25.0	3.4	4.0	4.1
Total		996	100	89.2	10.8	56.1.5	39.1	4.8		1343	100	88.7	11.3	42.5	27.2	30.3
	χ^2^(2) = 407.93^∗∗∗^			χ^2^(2) = 161.52^∗∗∗^		χ^2^(4) = 21.27^∗∗∗^			χ^2^(2) = 711.65^∗∗∗^			χ^2^(2) = 788.68^∗∗∗^		χ^2^(4) = 229.57^∗∗∗^		


*^∗∗∗^*p* < 0.001; ^*a*^Each raw adds up to 100% approximately; ^*b*^Each column adds up to 100% approximately; m, males; f, females; (M), mean rank or mean percentage.*

### Total Nominations in Terms of Major Areas of Creative Achievement

**Table 2** shows that in 2013 over half of (54.3%) the nominees were from the aesthetic areas whereas 45.1% were from the meritorious areas, χ^2^(2) = 407.93, *p* < 0.001. This difference was even more obvious in 2017, with 58.6% nominees from the aesthetic areas and 37.5% from the meritorious areas, χ^2^(2) = 711.65, *p* < 0.001. Thus, the total nomination also demonstrates a clear aesthetic salience.

### Total Nominations in Terms of Gender

In 2017, males accounted for 89.2% of the total nominees, χ^2^(2) = 161.52, *p* < 0.001 and this male dominance maintained in 2017, with 88.7% of the total nominees were males, χ^2^(2) = 788.68, *p* < 0.001. Absolutely male-dominated fields, according to the current study, are military and culinary fields (100% males in both years) and the scientific or inventive fields (99.2 and 99.5% males in 2013 and 2017, respectively).

### Total Nominations in Terms of the Nomination Order

In order to examine the participants’ intuitive preference in the nomination process, χ^2^ tests were conducted using a 3 (nomination orders) × 3 (areas of achievement) cross-tabulation analysis. The associations between these two variables were signification for in both years, χ^2^(4) = 21.27, Cramer’s *V* = 0.10, *p* = 0.000 in 2013 and χ^2^(4) = 229.57, Cramer’s *V* = 0.14, *p* = 0.000. In 2013, the odds ratios between 1st and 2nd, 1st and 3rd and 2nd and 3rd nomination in favor of the aesthetic salience areas are 1.11, 1.42, and 1.28 respective. The results for 2017 are 1.07, 1.12, and 1.04. Indeed, the participants were more likely to nominate creators from aesthetic salience areas in an earlier nomination than in a later nomination.

Taken together, the descriptive and cross-tab analyses of the nomination data revealed consistent patterns for the two points of measurement. They tended to nominate creative representatives from the aesthetic salience areas, particularly in their earlier nominations, and they were much more likely to nominate male rather than female creators.

### Personal and Social Attributes of the Nomination

**Table 3** presents the descriptive statistics and correlations between the variables for 2013 and 2017. From this table we can see a positive correlation between the gender of the participants and the gender of the nominated creators (*r* = 0.140 in 2013, *r* = 0.162 in 2017, *p* < 0.01 for both), indicating that people are more likely to nominate creators of the same gender. Female (*r* = -0.078, *p* < 0.05) and elder people (*r* = -0.078, *p* < 0.05), in comparison to male and younger people, tended to like the creators they nominated more. Older people also tended to rate the creativity (*r* = 0.072, *p* < 0.05) and SC (*r* = 0.107, *p* < 0.01) of the persons they nominated higher, but this was only found from the data of 2013. In both years, male creators were rated more creative than female creators (*r* = -0.223 in 2013, *r* = -0.151 in 2017, *p* < 0.01 for both) and the creators nominated in 2013 were also scored higher in SC (*r* = -0.100, *p* < 0.01). In 2017, no significant correlations were observed between the gender of the creators and liking, SC and SA. But the correlation between the gender of the creators and the creative fame was significant (*r* = -0.106, *p* < 0.01).

**Table 3:** Descriptive statistics and correlations between the variables: 2013 and 2017.

	*M*		*SD*	1	2	3	4	5	6	
**2013**										
1. Sex_P	–		–	1						
2. Age_P	28.28		9.88	-0.07^∗^	1					
3. Sex _C	–		–	0.14^∗∗^	0.01	1				
4. Creativity^a^	1.55		0.52	-0.02	0.07^∗^	-0.22^∗∗^	1			
5. SC^a^	1.70		0.61	0.01	0.11^∗∗^	-0.10^∗∗^	0.19^∗∗^	1		
6. Fame^b^	1.00		0.76	-0.05	-0.03	-0.03	0.12^∗∗^	0.29^∗∗^	1	

*^∗^*p* < 0.05; ^∗∗^p < 0.001; ^∗∗∗^; Sex_P: sex of the participants; Age_P: age of the participants; Sex_C: sex of the nominated person; SC: social contribution; ^*a*^Transformed data using SQRT(K-X); ^*b*^Transformed data using LG10(X).*										

	***M***	***SD***	**1**	**2**	**3**	**4**	**5**	**6**	**7**	**8**

**2017**										
1. Sex_P –	–	1							
2. Age_P	31.42	10.56	-0.10^∗∗^	1						
3. Sex _C	–	–	0.16^∗∗^	-0.02	1					
4. Creativity^a^	1.60	0.39	-0.05	0.04	-0.15**	(0.65)				
5. Liking^a^	1.90	0.41	-0.08^∗^	0.09^∗∗^	0.05	0.44^∗∗^	(0.76)			
6. SC^a^	1.97	0.51	-0.03	-0.02	-0.01	0.39^∗∗^	0.41^∗∗^	(0.82)		
7. SA^a^	1.98	0.40	0.01	0.00	-0.02	0.39^∗∗^	0.45^∗∗^	0.42^∗∗^	(0.78)	
8. Fame^b^	0.97	0.70	-0.01	-0.10^∗^	-0.11**	0.21^∗∗^	-0.05	0.33^∗∗^	0.28^∗∗^	1


*^∗^*p* < 0.05; ^∗∗^*p* < 0.001; Gender_P: Gender of the participants; Age_P: age of the participants; Gender_C: Gender of the nominated creator; SC: social contribution; SA: social acceptance; ^*a*^Transformed data using SQRT(K-X); ^*b*^Transformed data using LG10(X).*

The four factors of the Creator Evaluation Scale were moderately correlated with *r* ranging from 0.386 to 0.448, *p* < 0.01, indicating a good discrimination among the variables. These variables were all positively correlated with creative fame except liking. The strength of the correlations, however, were not very high, *r* = 0.207 for creativity, 0.328 for SC, and 0.278 for SA, *p* < 0.01 for all. Results of the descriptive statistics and correlations among the variables are presented in **Table 3**.

### The Mediating Effect of Social Contribution Between Creativity and Creative Fame: 2013

**FIGURE 1:**
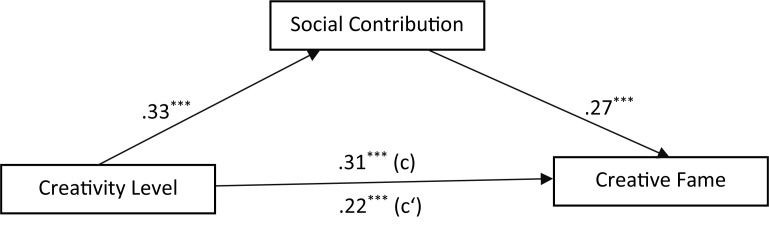
Mediation model of social contribution between creativity level and creative fame: aesthetic salience areas. ^∗∗∗^*p* < 0.001.

A previous study with Chinese students discovered a moderating effect of SC between creativity level and the creative fame (Cheung and Yue, 2007). However, the current study was not able to confirm this moderation model. Rather, a significant mediating effect of SC was found for both the nominations of the aesthetic salience areas and those of the meritorious salience areas. Higher rating of creativity leads to higher rating of SC (*b* = 0.33, *p* = 0.000), which increases the frequencies of nomination (creative fame) (*b* = 0.27, *p* = 0.000). The total effect of creativity on creative fame was significant, *c* = 0.31, *p* < 0.001. The effect of creativity on creative fame via SC is also significant, but with a lower *c’* of 0.22, *p* < 0.001. A bias-based bootstrap confidence interval (CI) for the indirect effect of creativity on creative fame via SC is significant, *ab* = 0.09, 95% IC (0.05, 0.14). The ratio of the indirect effect to the total effect is 0.29. **Figure 1** depicts this model.

**FIGURE 2:**
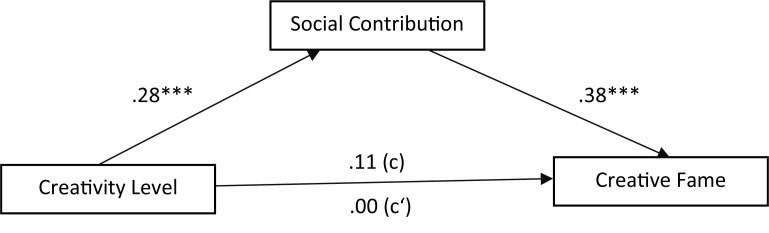
Mediation model of social contribution between creativity level and creative fame: meritorious salience areas. ^∗∗∗^*p* < 0.001.

Analysis of nominations from the meritorious salience areas revealed the similar pattern of mediation (**Figure 2**). Higher rating of creativity leads to higher rating of SC (*b* = 0.28, *p* = 0.000), which increases the frequencies of nomination (creative fame) (*b* = 0.38, *p* = 0.000). The total effect of creativity on creative fame was not significant, *c* = 0.11, *p* = 0.17. The effect of creativity on creative fame via SC is also not significant but with a lower *c’* of 0.00, *p* = 0.98. A bias-based bootstrap CI for the indirect effect of creativity on creative fame via SC is significant, *ab* = 0.11, 95% IC (0.05, 0.18). The ratio of the indirect effect to the total effect is 0.98.

In sum, this round of analysis shows that SC mediates the positive effect of creativity on creative fame and this mediating effect is stronger for the nominations from the meritorious salience areas than those from the aesthetic salience areas.

### The Mediating Effect of Social Contribution and Social Acceptance Between Creativity and Creative Fame: 2017

**FIGURE 3:**
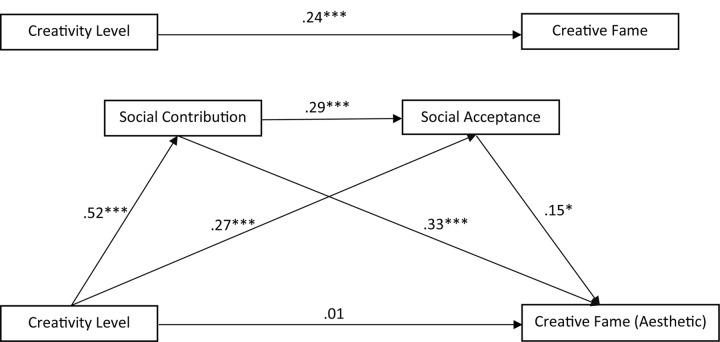
Three-path mediation model between creativity, SC, SA and creative fame: aesthetic areas. ^∗^*p* < 0.05, ^∗∗∗^*p* < 0.001

**FIGURE 4:**
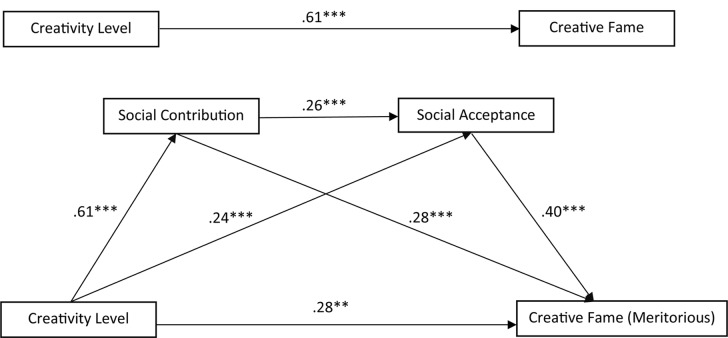
Three-path mediation model between creativity, SC, SA and creative fame: meritorious areas. ^∗∗^*p* < 0.01, ^∗∗∗^*p* < 0.001

The previous correlation analysis shows that creativity, SCs and SA are all significantly correlated with each other and each correlated significantly with creative fame, whereas liking was not significantly related to creative fame. Therefore, liking was excluded from the subsequent regression analysis. A three-path mediated effect was tested because of its advantage of being able to isolate the indirect effect of both mediators and meanwhile also allows to investigate the indirect effect passing through both of these mediators in a series (Taylor et al., 2008). Age and the gender of the creative icons were entered into the model as covariates, because both variables were correlated significantly with creative fame (see **Table 3**). The three-path mediated effect with age and the gender of the creative icons as covariates was tested for the nominations from the aesthetic (**Figure 3**) and meritorious (**Figure 4**) areas separately to detect any possible differences.

**Figure 3** shows that while nominating creative icons from the aesthetic areas, a total effect of creativity on the nomination results is significant, *b* = 0.24, SE = 0.06, *t*(759) = 4.11, *p* = 0.000. But this effect was consistently reduced via the indirect effect of SC or SA or both SC and SA. A significantly indirect effect of creativity on creative fame via SC was found, a_1_b_1_ = 0.17, 95% CI (0.11, 0.24). The indirect effect of creativity on creative fame via SA was also significant, a_2_b_2_ = 0.04, 95% CI (0.01, 0.08), though not that pronounced. In comparison to the above two-path mediator effect, the three-path mediator effect (creativity → SC → SA → creative fame) was even less pronounced but still significant, a_1_a_3_b_2_ = 0.02, 95% CI (0.004, 0.043). This means that though the three-path mediator model was significant, the two-path mediator models taking SC and SA separately into consideration can explain the nomination results better.

A very similar pattern of results was found for the nominations from the meritorious salience areas. The total effect of creativity on the nomination results was significant, *b* = 0.61, SE = 0.05, *t*(485) = 11.95, *p* = 0.000. The indirect effect of creativity on creative fame via SC was significant, a_1_b_1_ = 0.17, 95% CI (0.08, 0.27). The indirect effect of creativity on creative fame via SA was also significant, a_2_b_2_ = 0.09, 95% CI (0.05, 0.15), though not that pronounced. In comparison to the above two-path mediator effect, the three-path mediator effect (creativity → SC → SA → fame) was even less pronounced but still significant, a_1_a_3_b_2_ = 0.06, 95% CI (0.03, 0.10). Like the analysis for the nominations from the aesthetic areas, the two-path mediator models taking SC and SA separately into consideration demonstrated bigger effect sizes. Overall, the two-path and three-path mediator effects of this model were stronger than the model of the aesthetic areas, indicating that the mediating effects of SC and SA are even stronger in nominating the creative icons from the meritorious areas.

To summarize, SC and SA each mediated the positive relation between creativity and creative fame. Further, the three-path mediation model of creativity → SC → SA → creative fame was also significant for both nomination conditions. Overall, the mediating effect of SC and SA was stronger for the nomination from the meritorious areas than the aesthetic areas.

## Discussion

Analysis of the nominations of 2013 and 2017 revealed a consistent pattern: German young adults predominantly nominated creators from the aesthetic salience field, especially the field of literature and arts as representatives of creativity in Germany. These results are in line with the previous studies (Yue et al., 2011; Tang and Moser, 2018). Historically, Germany is well acknowledged as a land of poets and thinkers due to the innovative contributions Germans made in arts, music, or literature in the last three centuries (Breuilly, 2001). The aesthetic salience discovered in the current study reflects this country image of Germany which evokes various spiritualized thinkers that strongly shaped German culture (Hohendahl and Franciscono, 1989). The availability heuristic, having easy access to information concerning creativity and innovation in Germany without searching exhaustively for alternatives (Tversky and Kahneman, 1973; Sherman and Corty, 1984), can help to explain this result. Besides, nominating these individuals appears to be influenced by certain social norms. “Social norms are rules and standards that are understood by members of a group, and that guide and/or constrain social behavior without the force of law.” (Cialdini and Trost, 1998, p. 152). Because of the widely appreciated contributions persons like Johann S. Bach and Ludwig van Beethoven made to the German culture, people tend to feel the necessity to fulfill this social norm to appreciate the contributions made by such individuals.

It is worth noting that though creators from the aesthetic salience fields dominate the results, Albert Einstein occupies the first place with an obvious lead in both years. This result reflects the impact of science in the fast-growing society. Science is a rational enterprise whose primary mission is to maximize objective value through systematic exploration (Habermas, 1981). It is the important impetus of economic growth in modern society. Germany is Europe’s biggest economy and is famed for its technological achievements. Important basis for such achievements is the German tradition of respecting scientists and encouraging prudent scientific endeavors. Einstein is a world-wide known icon of science. No wonder he was nominated in both years as the greatest creative icon. Although Albert Einstein is mostly recognized for his scientific discoveries, he also stands out for his rebellious and non-conforming behavior especially in the field of politics and militarism. These insurgent manners are often attributed to creative persons (Eysenck, 1995; Fürst et al., 2014). which could be another explanation why Albert Einstein was nominated in both studies.

A surprising result of the top 10 nominees was Angela Merkel. The study of Yue et al. (2011) showed that German undergraduates mostly nominated philosophers, artists, and writers but rarely nominated politicians. Another study revealed that the nominated German creators were disproportionately male (Tang and Moser, 2018). Merkel is “twice exceptional” because of her being a female and a politician, which theoretically should dramatically decrease the possibility of being nominated as a creative icon. Growing from a quantum chemist of the former East Germany into the most powerful woman in the world, Merkel’s political career is full of surprise and unexpectedness. Surprise is commonly regarded as one indispensable component of people’s perception of creativity (Bruner, 1962; Acar et al., 2017). On her way to become not only the first female chancellor of Germany but also the longest-serving incumbent head of government in the European Union, Merkel possesses personal traits that creative individuals usually have such as strong intrinsic motivation (Golann, 1963; Amabile, 1996) and efforts and willingness to grow (McCrae, 1987).

The fact that the aesthetic salience was more pronounced with the earlier than the later nomination can be explained by the two-system approach of thinking proposed by Kahneman (2011). According to this theory, two basic systems of thinking function and influence our judgment and choice. Whereas System 1 operates automatically, quickly, and intuitively, with little or no effort, System 2 operates more controlled, effortful and deliberate. In making judgments, such as nominating creative persons, System 1 will first be activated to name persons without much effort. Because of the great importance that the Western culture attaches to the aesthetic attributes of creativity (Rump, 1982; Sternberg, 1985; Helson, 1988) and the expression of one’s individuality (Sternberg, 1985; Runco and Bahleda, 1987), creators from the aesthetic salience fields become their first, intuitive choice. After exhausting System 1, System 2 has to be consulted to continue with the further nomination. In this case, the subsequent judgments or choices will become more controlled and effortful, where might also include the consideration of the social factors related to the creators.

In both years, male creators accounted for almost 90% of the total creative nominees, suggesting a male-dominance in people’s perception of creativity at the highest level. This results echoes the results of numerous studies (Piirto, 1991; Simonton, 1994; Cheung and Yue, 2007; Thompson, 2016; Tang and Moser, 2018). One direct reason for this gender disparity lies in the fact that women are overall underrepresented in science, technology, engineering, and mathematics (STEM) fields (Blickenstaff, 2005) as well as the non-STEM fields (such as arts) where innate abilities instead of efforts are regarded as more important (Meyer et al., 2015). In addition, the less productivity of female creators in comparison to male creators (Baer and Kaufman, 2008) can further reduce the recognizability of the creative achievements of women, as there is evidence of a high correlation between quantity and quality of creative achievements (Kim, 2006). The underrepresentation and unproductivity of women at the eminence level, however, has deep historical, cultural, and social reasons. In old days, women were deprived of equal rights of education and of property ownership. As consequences, their talents were either not fully developed or, if they had achieved something exceptional, the merits were given to their husbands or fathers (Nochlin, 1988; Bellis, 2017). In most countries many people are ready to accept that a woman’s “real” achievement is defined in terms of motherhood and nurturance (Kerr, 1997). But the creative products or solutions that women have created in their motherhood and nurturance are regarded as minor in our modern societies where creative fields are still dominate by men and the evaluation criteria of creativity are mainly set according to men’s perceptions and preferences. This makes women creative achievements usually not valued or devalued. Meanwhile, the role congruity theory (Eagly and Karau, 2002) implies that cultural values and social roles imposed to men and women force people of both genders to conform to their gender roles, leading men and woman develop different goals, which in turn shape and socialize gender-roles and self-images. Gender socialization already happens in early childhood. Research revealed that elementary school children gain more popularity, if they fulfill stereotypical gender roles in terms of masculinity and femininity (Adler et al., 1992). As consequences of gender roles, women’s creative engagement and achievements are usually discouraged, disrespected and underestimated (Tang, 2010). Considering the fact that literature reviews or meta-analyses actually fail to provide empirical support to the widely shared belief of an overall male lead in creativity (Baer and Kaufman, 2008; Ma, 2009; Thompson, 2016), the under-estimation of female creators deserves more attention from researchers, educators, and politicians. It is a waste of human intelligence and creativity if the creative field is dominated by male contributions. More cultural and institutional support is needed to amend the “leady pipeline” in the developmental path of creative women.

In both years’ studies, SC turned out to be a significant mediator between creativity and creative fame. The mediation model was extended to include SA in 2017. Like SC, SA also mediated the relationship between creativity and creative fame. In addition, the three-path serial mediation model of SC and SA was also significant in both the aesthetic and meritorious salience cases. This means the personal and social attributes act in a sequential way to predict the frequency of nomination in that higher creativity level leads to higher rating in SC, which leads to higher rating of SA, which in turn leads to the nomination. The positive relationship between creativity and SC, in terms of personal and social achievements, has been empirically proven through longitudinal studies. For example, a 40-year follow-up study of the Torrance Center found that people’s creativity measured by Torrance Test of Creative Thinking 40 years ago was able to explain 23% of the variance in creative achievements 40 years later (Cramond et al., 2005). Their 50-year follow-up study also found that an interaction of intelligence and creativity was significantly related to public achievement (Runco et al., 2010). The higher the SC of a person is, the more likely the person will be accepted by society as a creative person, as the social psychological theory of creativity requires that creativity exists only if it leads to concrete products that consensually assessed by experts as creative (Amabile, 1996). SA facilitates the process of being nominated, as individuals usually rely on the observation of others to adapt their behaviors, revise their judgments, or make decisions (Couzin et al., 2011).

The insignificant correlation between liking and the creative fame shows that participants can still appreciate the creative contributions of a creator although they dislike the person. This ambivalent reaction can be due to the complex personality traits of creative persons. In general, it is known that creative persons are not easy to handle. Csikszentmihalyi (1996) points out that creative people have a complex personality, holding various contradictory extremes, for example, being extroverted and introverted at the same time. Not only positive personality characteristic can be found among creative individuals. Highly creative personalities also share some “dark” traits that are not necessarily desirable for normal people, such as being depressed (Ludwig, 1995), psychopathic (Galang et al., 2016), narcissistic (Furnham et al., 2013), potentially dishonest (Gino and Ariely, 2012) and having more biological vulnerability and negative emotions (particularly among creative artists) (Akinola and Mendes, 2008). These unpleasant personality characteristics of creative persons can trigger negative emotional reaction such as the low level of liking.

### Limitations and Future Studies

The following limitations need to be noted: First, the whole study was grounded on the differentiation of meritorious vs. aesthetic salience areas theorized through studies using Chinese samples (Yue, 2003; Yue et al., 2011), but the validity of this theory has not yet been examined. In cross-validating a creativity domain questionnaire developed in the United States for the Chinese context, we found that the Chinese factorial structure of the domain was somewhat different from that of the American (Werner et al., 2014). Germany is part of the Western culture, so the German perception of domains can quite possibly differ from the Chinese. Hence, it is recommendable that the internal consistency, factorial validity as well as convergent and divergent validity of the meritorious vs. aesthetic salience areas should be systematically examined.

Second, accessibility heuristic (Tversky and Kahneman, 1973) and affect heuristic (Slovic et al., 2007) cover both the cognitive and affect aspects of people’s judgments and decision making, which have great potential to help us probe the underlying mechanisms of nomination of creators. Due to the scope of the current study, these theories were only applied to justify the inclusion of variables (e.g., order of nomination and liking) and to explain their influence on German people’s perception of creative representatives. Future studies can consider applying the accessibility and affect heuristic theories in experimental settings to more closely examine the psychological mechanism of nomination.

Third, SC was found mediating the relationship between creativity and creative fame in the current study. This result is inconsistent with the previous study, which found a moderation effect of SC (Cheung and Yue, 2007). In their classic work about moderator vs. mediator variable distinction in social psychological research, Baron and Kenny (1986) postulate that though mediators are typically applied to explain underling mechanism between a stimuli and response or input and output, it is also quite often to have group-level mediators such as norms, group think or cohesiveness as intervening process in social psychology. That is, both the mediation and moderation effect of SC between creativity and creative fame are plausible. As both studies were conducted in different countries, we can only assume that the different role that social factors play in people’s perceptions of creative icons might be due to the influence of different cultures. To test this hypothesis, cross-cultural studies are needed.

## Conclusion

Using a time series design, the current study serves as the first study of the kind to detect the temporal pattern of the nomination of creative icons with a time interval of 4 years. Consistent patterns were found through the analysis over time: The aesthetic salience is obvious and male creators dominate the nominee lists in both years. Einstein ranks the first in both lists followed by Goethe and Merkel. Merkel is the only female nominee in both Top 10 lists and overall, only about 10% of the nominees are females. In answering the question “Where are all the female geniuses?,” Upson and Friedman (2012) pointed out, “Women tend to choose work-life balance rather than the pursuit of eminence – although the choice is not entirely freely made” (p. 63). Two streams of studies can be extremely beneficial to help us understand this phenomenon: investigation of gender differences through the interactions among aptitudes, motivations, and opportunities and studies of changes over time in situations where gender bias has been reduced (Baer and Kaufman, 2008). In both years, the SC and SA mediate the positive relation between creativity and creative fame and the mediating effect of both social factors is stronger for the nomination from the meritorious than the aesthetic areas. This result confirms the relevance and importance of the sociocultural approaches to creativity (for a review, see Sawyer, 2012; Tang, 2017), which provide very promising diagrams for studying creativity across domains and cultures.

## Ethics Statement

This study was carried out in accordance with the recommendations of the Guidelines for Scientific Research, Germany. The protocol was approved by the Institutional Review Board (IRB) of the University of Applied Management, Germany. The consent of the participants was obtained by virtue of survey completion after they were provided with sufficient information about the study, including the purpose of the study.

## Author Contributions

MT led the research design and collected and analyzed the data. CW joined the research design, organized the coding, and assisted in the interpretation of the results. SH brought new insights and imbedded new literature in revising the paper.

## Conflict of Interest Statement

The authors declare that the research was conducted in the absence of any commercial or financial relationships that could be construed as a potential conflict of interest.

## Footnotes

1 It is worth noting that this dimension was named “influence of others” in the previous study (Tang and Moser, 2018).

## References

[B1] Acar S. Burnett C. Cabra J. F. (2017). Ingredients of creativity: originality and more. *Creat. Res. J.* 29 133–144. 10.1080/10400419.2017.1302776

[B2] Adler P. Kless S. Adler P. (1992). Socialization to gender roles: popularity among elementary school boys and girls. *Sociol. Educ.* 65 169–187. 10.2307/2112807

[B3] Ajzen I. Fishbein M. F. (1980). *Understanding Attitudes and Predicting Social Behavior.* Englewood Cliffs, NJ: Prentice-Hall.

[B4] Akinola M. Mendes W. B. (2008). The dark side of creativity: biological vulnerability and negative emotions lead to greater artistic creativity. *Pers. Soc. Psychol. Bull.* 34 1677–1686. 10.1177/0146167208323933 18832338 PMC2659536

[B5] Amabile T. M. (1996). *Creativity in Context: Update to the Social Psychology of Creativity.* Boulder, CO: Westview.

[B6] Baer J. Kaufman J. (2008). Gender differences in creativity. *J Creat Behav.* 42 75–105. 10.1002/j.2162-6057.2008.tb01289.x

[B7] Baron R. M. Kenny D. A. (1986). The moderator–mediator variable distinction in social psychological research: conceptual, strategic, and statistical considerations. *J. Pers. Soc. Psychol.* 51 1173–1182. 10.1037/0022-3514.51.6.1173 3806354

[B8] Bellis M. (2017). *How Many Women Inventors Are There? ThoughtCo.* Available at: https://www.thoughtco.com/how-many-women-inventors-are-there-1992649

[B9] Blickenstaff J. C. (2005). Women and science careers: leaky pipeline or gender filter? *Gender Educ.* 17 369–386. 10.1080/09540250500145072

[B10] Breuilly J. (2001). *19th Century Germany: Politics, Culture and Society 1780–1918.* London: Hodder Arnold.

[B11] Bruner J. S. (1962). “The conditions of creativity,” in *Contemporary Approaches to Cognition*, eds Gruber H. Terrell G. Wertheimer M. (New York, NY: Atherton), 1–30.

[B12] Carson S. H. Peterson J. B. Higgins D. M. (2005). Reliability, validity, and factor structure of the creative achievement questionnaire. *Creat. Res. J.* 17 37–50. 10.1207/s15326934crj1701_4 24597441

[B13] Chan D. W. Chan L. (1999). Implicit theories of creativity: teachers’ perception of student characteristics in Hong Kong. *Creat. Res. J.* 12 185–195. 10.1207/s15326934crj1203_3

[B14] Cheung C. Yue X. D. (2007). Which Chinese creators are famous and why: views from Hong Kong and Mainland Chinese students. *J. Creat. Behav.* 41 177–196. 10.1002/j.2162-6057.2007.tb01287.x

[B15] Cialdini R. B. Trost M. R. (1998). “Social influence: Social norms, conformity and compliance,” in *The Handbook of Social Psychology*, eds Gilbert D. T. Fiske S. T. Lindzey G. (New York, NY: McGraw-Hill), 151–192.

[B16] Couzin I. Ioannou C. Demirel G. Gross T. Torney C. J. Hartnett A. (2011). Uninformed individuals promote democratic consensus in animal groups. *Science* 334 1578–1580. 10.1126/science.1210280 22174256

[B17] Cramond B. Matthews-Morgan J. Bandalos D. Zuo L. (2005). A report on the 40-year follow-up of the torrance tests of creative thinking: alive and well in the new millennium. *Gift. Child Q.* 49 283–291. 10.1177/001698620504900402

[B18] Csikszentmihalyi M. (1994). “The domain of creativity,” in *Changing the World*, eds Feldman D. H. Csikszentmihalyi M. Gardner H. (Westport, CT: Praeger), 135–158.

[B19] Csikszentmihalyi M. (1996). *Creativity Flow and the Psychology of Discovery and Invention.* New York, NY: Harper Collins.

[B20] Dineen R. Niu W. (2008). The effectiveness of Western creativity teaching methods in China: an action research project. *Psychol. Aesthetics Creat. Arts* 2 42–52. 10.1037/1931-3896.2.1.42

[B21] Eagly A. H. Karau S. J. (2002). Role congruity theory of prejudice toward female leaders. *Psychol. Rev.* 109 573–598. 10.1037/0033-295X.109.3.573 12088246

[B22] Eysenck H. J. (1995). *Genius: The Natural History of Creativity.* Cambridge: Cambridge University Press 10.1017/CBO9780511752247

[B23] Furnham A. Hughes D. J. Marshall E. (2013). Creativity, OCD, Narcissism and the Big Five. *Think. Skills Creat.* 10 91–98. 10.1016/j.tsc.2013.05.003

[B24] Fürst G. Ghisletta P. Lubart T. (2014). Toward an integrative model of creativity and personality: theoretical suggestions and preliminary empirical testing. *J. Creat. Behav.* 50 87–108. 10.1002/jocb.71

[B25] Galang A. J. R. Castelo V. L. C. Santos L. C. Perlas C. M. C. Angeles M. A. B. (2016). Investigating the prosocial psychopath model of the creative personality: evidence from traits and psychophysiology. *Pers. Individ. Dif.* 100 28–36. 10.1016/j.paid.2016.03.081

[B26] Gino F. Ariely D. (2012). The dark side of creativity: original thinkers can be more dishonest. *J. Pers. Soc. Psychol.* 102 445–459. 10.1037/a0026406 22121888

[B27] Glãveanu V. P. (2011). Is the light bulb still on? Social representations of creativity in a Western context. *Int. J. Creat. Prob. Solving* 21 53–72.

[B28] Golann S. E. (1963). Psychological study of creativity. *Psychol. Bull.* 60 548–565. 10.1037/h0041573 14081204

[B29] Habermas J. (1981). *The Theory of Communicative Action: Reason and the Rationalization of Society*, Vol. 1. Cambridge: Polity Press.

[B30] Hass R. W. Reiter-Palmon R. Katz-Buonincontro J. (2017). “Are implicit theories of creativity domain specific? Evidence and implications,” in *The Creative Self: Effect of Beliefs, Self-efficacy, Mindset, and Identity*, eds Karwowski M. Kaufman J. C. (San Diego, CA: Elsevier Academic Press), 219–234.

[B31] Helson R. (1988). “The creative personality,” in *Innovation: A Cross Disciplinary Perspective*, eds Gronhaug K. Kaufmann G. (Oslo: Norwegian University Press), 29–64.

[B32] Hohendahl P. Franciscono R. (1989). *Building a National Literature: The Case of Germany, 1830–1870.* London: Cornell University Press.

[B33] Kahneman D. (2011). *Thinking, Fast and Slow.* London: Allen Lane.

[B34] Karwowski M. (2009). I’m creative, but am I Creative? Similarities and differences between self-evaluated Small and Big-C creativity in Poland. *Int. J. Creat. Prob. Solving* 19 7–26.

[B35] Kerr B. A. (1997). *Smart girls: a new psychology of girls, women, and giftedness.* Scottsdale, AZ: Psychology Press.

[B36] Kim K. H. (2006). Can we trust creativity tests? A review of the Torrance Tests of Creative Thinking (TTCT). *Creat. Res. J.* 18 3–14. 10.1207/s15326934crj1801_2

[B37] Kogan N. (1974). Creativity and sex differences. *J. Creat. Behav.* 8 1–14. 10.1002/j.2162-6057.1974.tb01103.x

[B38] Lan L. Kaufman J. C. (2012). American and Chinese similarities and differences in defining and valuing creative products. *J. Creat. Behav.* 46 285–306. 10.1002/jocb.19

[B39] Lim W. Plucker J. A. (2001). Creativity through a lens of social responsibility: implicit theories of Creativity with Korean samples. *J. Creat. Behav.* 35 115–130. 10.1002/j.2162-6057.2001.tb01225.x

[B40] Lubart T. I. (1999). “Creativity across cultures,” in *Handbook of Creativity*, ed. Sternberg R. J. (Cambridge: Cambridge University Press), 339–350.

[B41] Ludwig A. M. (1995). *The Price of Greatness.* New York, NY: Guilford Press.

[B42] Luksyte A. Unsworth K. L. Avery D. (2018). Innovative work behavior and sex-based stereotypes: examining sex differences in perceptions and evaluations of innovative work behavior. *J. Organ. Behav.* 39 292–305. 10.1002/job.2219

[B43] Ma H. (2009). The effect size of variables associated with creativity: a meta-analysis. *Creat. Res. J.* 21 30–42. 10.1080/10400410802633400

[B44] McCrae R. R. (1987). Creativity, divergent thinking, and openness to experience. *J. Pers. Soc. Psychol.* 52 1258–1265. 10.1037/0022-3514.52.6.1258

[B45] Meyer M. Cimpian A. Leslie S.-J. (2015). Women are underrepresented in fields where success is believed to require brilliance. *Front. Psychol.* 6:235. 10.3389/fpsyg.2015.00235 25814964 PMC4356003

[B46] Moussaïd M. Kämmer J. E. Analytis P. P. Neth H. (2013). Social influence and the collective dynamics of opinion formation. *PLoS One* 8:e78433. 10.1371/journal.pone.0078433 24223805 PMC3818331

[B47] Nochlin L. (1988). *Women, Art and Power and Other Essays.* Boulder, CO: Westview Press.

[B48] Paletz S. B. Peng K. (2008). Implicit theories of creativity across cultures: novelty and appropriateness in two product domains. *J. Cross Cult. Psychol.* 39 286–302. 10.1177/0022022108315112

[B49] Piirto J. (1991). Why are there so few? (Creative women: Visual artists, mathematicians, musicians). *Roeper Rev.* 13 142–147. 10.1080/02783199109553340

[B50] Piirto J. (2004). *Understanding Creativity.* Scottsdale, AZ: Great Potential Press.

[B51] Plucker J. A. Lim W. Lee K. (2017). Viewing through one prism or two? Discriminant validity of implicit theories of intelligence and creativity. *Psychol. Aesthetics Creat Arts* 11 392–402. 10.1037/aca0000126

[B52] Proudfoot D. Kay A. C. Koval C. Z. (2015). A gender bias in the attribution of creativity: archival and experimental evidence for the perceived association between masculinity and creative thinking. *Psychol. Sci.* 26 1751–1761. 10.1177/0956797615598739 26386015

[B53] Rump E. E. (1982). Relationships between creativity, arts orientation, and esthetic-preference variables. *J. Psychol.* 110 11–20. 10.1080/00223980.1982.9915320

[B54] Runco M. A. (1989). Parents’ and teachers’ ratings of the creativity of children. *J. Soc. Behav. Pers.* 4 73–83.

[B55] Runco M. A. Bahleda D. (1987). Implicit theories of artistic, scientific and everyday creativity. *J. Creat. Behav.* 20 93–98. 10.1002/j.2162-6057.1986.tb00423.x

[B56] Runco M. A. Johnson D. (2002). Parents’ and teachers’ implicit theories of children’s creativity: a cross-cultural perspective. *Creat. Res. J.* 14 427–438. 10.1207/S15326934CRJ1434_12

[B57] Runco M. A. Johnson D. J. Bear P. K. (1993). Parents’ and teachers’ implicit theories of children’s creativity. *Child Study J.* 23 91–113.

[B58] Runco M. A. Millar G. Acar S. Cramond B. (2010). Torrance Tests of Creative Thinking as predictors of personal and public achievement: a fifty-year follow-up. *Creat. Res. J.* 22 361–368. 10.1080/10400419.2010.523393

[B59] Sawyer R. K. (2012). *Sociocultural Approaches. Explaining Creativity; The Science of Human Innovation*, 2nd Edn. New York: NY: Oxford University Press, 211–229.

[B60] Schwartz B. (1998). Postmodernity and historical reputation: Abraham Lincoln in late twentieth-century America memory. *Soc. Forces* 77 63–103. 10.1093/sf/77.1.63

[B61] Seng Q. K. Keung H. K. Cheng S. K. (2008). Implicit theories of creativity: a comparison of student-teachers in Hong Kong and Singapore. *Compare J. Comp. Educ.* 38 71–86. 10.1080/03057920701419959

[B62] Sewell D. K. (2018). Heterogeneous susceptibilities in social influence models. *Soc. Netw.* 52 135–144. 10.1016/j.socnet.2017.06.004

[B63] Sherman S. J. Corty E. (1984). “Cognitive heuristics,” in *Handbook of Social Cognition* Vol. 1 eds Wyer R. S. Srull T. K. (Hillsdale, NJ: Lawrence Erlbaum), 189–286.

[B64] Simonton D. K. (1988). “Creativity, leadership, and chance,” in *The Nature of Creativity*, ed. Sternberg R. J. (New York, NY: Cambridge University Press), 386–426.

[B65] Simonton D. K. (1994). *Greatness: Who Makes History and Why.* New York, NY: Guilford Press.

[B66] Slovic P. Finucane M. L. Peters E. MacGregor D. G. (2007). The affect heuristic. *Eur. J. Oper. Res.* 177 1333–1352. 10.1016/j.ejor.2005.04.006

[B67] Smith C. D. Wright L. (2000). Perceptions of genius: einstein, Lesser Mortals and Shooting Stars. *J. Creat. Behav.* 34 151–164. 10.1002/j.2162-6057.2000.tb01208.x

[B68] Sparrow B. Liu J. Wegner D. M. (2011). Google Effects on memory: cognitive consequences of having information at our fingertips. *Science* 333 776–778. 10.1126/science.1207745 21764755

[B69] Spiel C. von Korff C. (1988). Implicit theories of creativity: the conceptions of politicians, scientists, artists and school teachers. *High Ability Stud.* 9 43–58. 10.1080/1359813980090104

[B70] Spinks J. A. Lam L. M. Lingen G. V. (1998). “Cultural determinants of creativity: An implicit theory approach,” in *Creative Thinking: Towards Broader Horizons*, ed. Dingli S. (Malta: University of Malta Press).

[B71] Sternberg R. J. (1985). Implicit theories of intelligence, creativity, and wisdom. *J. Pers. Soc. Psychol.* 49 607–627. 10.1037/0022-3514.49.3.607

[B72] Tabachnick B. G. Fidell L. S. (2007). *Using Multivariate Statistics*, 5th Edn. Boston, MA: Allyn & Bacon.

[B73] Tang M. (2010). *China’s Young Inventors: A Systematic View of the Individual and Environmental Factors.* Doctoral dissertation, Faculty of Psychology and Educational Sciences, München.

[B74] Tang M. (2017). “Creativity and innovation: Basic concepts and approaches,” in *Handbook of the Management of Creativity and Innovation: Theory and Practice*, eds Tang M. Werner C. H. (Singapore: World Scientific Press).

[B75] Tang M. Moser M. (2018). “Nomination of domestic and overseas creative celebrities: The German style and the factors behind it,” in *Handbook of Social Creativity Research*, eds Lebuda I. Glaveanu V. P. (New York, NY: Palgrave).

[B76] Taylor A. B. MacKinnon D. P. Tein J.-Y. (2008). Tests of the three-path mediated effect. *Organ. Res. Methods* 11 241–269. 25316269

[B77] Thompson T. L. (2016). *The Mothers and Fathers of Invention: A Meta-Analysis of Gender Differences in Creativity.* Available at: http://purl.flvc.org/fsu/fd/FSU_2016SP_Thompson_fsu_0071E_13059

[B78] Tversky A. Kahneman D. (1973). Availability: a heuristic for judging frequency and probability. *Cogn. Psychol.* 5 207–232. 10.1016/0010-0285(73)90033-9

[B79] Upson S. Friedman L. F. (2012). Where are all the female geniuses? *Sci. Am. Mind* 23 63–65. 10.1038/scientificamericanmind1112-63

[B80] Werner C. Tang M. Kruse J. Kaufman J. Spörrle M. (2014). The chinese version of the revised creativity domain questionnaire (CDQ-R): first Evidence for its Factorial Validity and Systematic Association with the Big Five. *J. Creat. Behav.* 48 254–275. 10.1002/jocb.51

[B81] Yue X. D. (2003). Meritorious attribution bias: how Chinese undergraduates perceive Chinese and foreign creators. *J. Creat. Behav.* 37 151–178. 10.1002/j.2162-6057.2003.tb00831.x

[B82] Yue X. D. (2004). However is influential is creative: how Chinese undergraduates choose creative people in Chinese societies. *Psychol. Rep.* 94 1235–1249. 10.2466/pr0.94.3c.1235-1249 15362398

[B83] Yue X. D. Bender M. Cheung C. K. (2011). Who are the best known national and foreign creators–a comparative study among undergraduates in China and Germany? *J. Creat. Behav.* 45 23–37. 10.1002/j.2162-6057.2011.tb01082.x

[B84] Yue X. D. Rudowicz E. (2002). Perception of the most creative Chinese by undergraduates in Beijing, Guangzhou, Hong Kong and Taipei. *J. Creat. Behav.* 36 88–104.

[B85] Zajonc R. B. (1980). Feeling and thinking: preferences need no inferences. *Am. Psychol.* 35 151–175. 10.1037/0003-066X.35.2.151

